# Challenges of copro‐parasitological surveys in wild Iberian ibex (*Capra pyrenaica*) populations addressed through a combination of molecular and statistical tools

**DOI:** 10.1002/ece3.10172

**Published:** 2023-06-06

**Authors:** Catarina Fontoura‐Gonçalves, Érica Portocarrero, Ana Oliveira, João Lozano, Laura Rinaldi, Giuseppe Cringoli, Luís Madeira de Carvalho, Nuno Santos

**Affiliations:** ^1^ Instituto de Ciências Biomédicas Abel Salazar (ICBAS) Universidade do Porto Porto Portugal; ^2^ Faculty of Veterinary Medicine, CIISA – Centre for Interdisciplinary Research in Animal Health University of Lisbon, Avenida da Universidade Técnica Lisbon Portugal; ^3^ Associate Laboratory for Animal and Veterinary Sciences (AL4AnimalS) Lisbon Portugal; ^4^ Department of Veterinary Medicine and Animal Production University of Napoli Federico II Naples Italy; ^5^ CIBIO, Centro de Investigação em Biodiversidade e Recursos Genéticos, InBIO Laboratório Associado Universidade do Porto Vairão Portugal; ^6^ BIOPOLIS Program in Genomics, Biodiversity and Land Planning CIBIO Vairão Portugal

**Keywords:** *Capra pyrenaica*, gastrointestinal parasites, molecular biology, N‐mixture models, site‐occupancy models

## Abstract

Copro‐parasitological surveys in wildlife face challenges due to the secretive nature of many species and the unknown performance of the diagnostic tests employed. To overcome these issues, we used a combination of hierarchical models (site‐occupancy and N‐mixture models) applied to copro‐parasitological data obtained from fecal samples assigned to the host species by molecular methods in the Iberian ibex in north‐western Iberian Peninsula. The aims were to compare the performance of four diagnostic tests (Mini‐FLOTAC, McMaster, Willis flotation, and natural sedimentation) and to use this methodological approach (molecular analysis with hierarchical models) to better estimate positivity proportion and shedding intensity in a wild ibex population. Pooled fecal samples were collected, and those confirmed by molecular analyses to be the host species in question were included in the study. Hierarchical models confirmed different performances of each diagnostic test, with Mini‐FLOTAC showing higher sensitivity for eimeriid coccidia, Willis flotation (for proportion positive) and McMaster (for shedding intensity) in gastrointestinal Strongylida, and equal performance of MiniFlotac/Willis flotation (for proportion positive) and MiniFlotac/McMaster (for shedding intensity) in *Moniezia* spp. This study employed a combination of molecular and statistical methods that improved the estimates of prevalence and shedding intensity and allowed us to compare the performance of four diagnostic tests while assessing the effect of covariates. Such improvements are critical to enhancing inference in non‐invasive wildlife copro‐parasitological studies.

## Graphical abstract

Parasitological survey of wild caprines in a transfrontier protected area in the Iberian Peninsula. Addressed through molecular techniques and applied hierarchical models to correct prevalence and shedding intensity by the imperfect test sensitivity. The aims were to compare the performance of four diagnostic tests while assessing the effect of covariates. Such methodological improvements are critical to enhancing inference in non‐invasive wildlife copro‐parasitological studies.

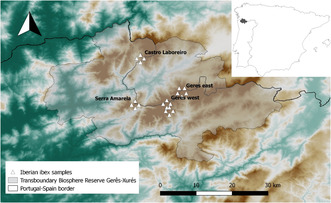

## 1 INTRODUCTION

Parasitological wildlife surveys face challenges not encountered with livestock (Ryser‐Degiorgis, 2013). The secretive nature of some species along with other challenges such as the presence of mixed‐species groups or aggressiveness are some of the obstacles to the collection of fresh samples (Rousseau et al., 2021). Ethical issues, associated with capturing and handling wildlife and its impact on the animals and the environment make non‐invasive samples preferable over invasive ones (Lindsjö et al., 2016). Non‐invasive fecal samples collected from the environment need to be assigned the correct host species; failing to do so can bias the prevalence estimates (Cardoso et al., 2021; Zhang et al., 2011). Field identification of fecal samples can be inaccurate (e.g., 18% inaccuracy in carnivore samples – Monterroso et al., 2019). One way to minimize the bias from non‐invasive sampling for copro‐parasitological surveys in wildlife is to identify the host species using fecal DNA (Brandell et al., 2022; Cardoso et al., 2021; Zhang et al., 2011).

Another challenge that parasitological surveys face is the imperfect and often unknown performance of the diagnostic methods in each host and parasite species (Abdu et al., 2022; Lachish et al., 2012). In fact, the diagnostic sensitivity of standard copro‐parasitological methods is unknown for many parasite–host systems. It thus cannot estimate true prevalence, achieved by correcting the apparent prevalence for the sensitivity of the diagnostic test (Lachish et al., 2012). Hierarchical models such as site‐occupancy (MacKenzie et al., 2002) and N‐mixture (Royle, 2004) were developed to overcome the same challenges when estimating the distribution and abundance of wildlife species and therefore can be applied to parasitological surveys. Using replicates of the diagnostic test applied to the same samples, hierarchical models can correct the estimated prevalence or abundance for the imperfect detection of the diagnostic method (Lachish et al., 2012). Particularly, site‐occupancy models can estimate the probability of detection (*p*), which corresponds to the diagnostic sensitivity in qualitative methods, while N‐mixture models can estimate the probability of detecting one parasite form in quantitative methods (Lachish et al., 2012; MacKenzie et al., 2002; Royle, 2004). Hierarchical models thus correct the estimated prevalence (site‐occupancy models) or abundance (N‐mixture models) for the imperfect detection of the diagnostic method (Lachish et al., 2012). Although inherently suited to wildlife parasitological survey data, these methods have rarely been used in this field (e.g., Zanet et al., 2017).

Here we apply site‐occupancy and N‐mixture models to copro‐parasitological data obtained from fecal samples assigned to the host species by molecular methods in an Iberian ibex population in north‐western Portugal. The Iberian ibex, *Capra pyrenaica*, is an Iberian Peninsula endemic species currently classified globally as ‘Least concern’ by The IUCN (Herrero et al., 2021) but in Portugal, it is considered as ‘Critically endangered’ (Cabral et al., 2005). The Gerês‐Xurés Transboundary Biosphere Reserve (GXTBR, 41°43′49.22″N 8°9′42.05″W, max elevation: 1546 m) currently holds the single population of Iberian ibex in Portugal, estimated in 2011–2012 at 576 individuals (CI_95_ 356–930) in four geographical nuclei: Gerês east, Gerês west, Serra Amarela, and Castro Laboreiro (Fonseca et al., 2017; Moço et al., 2006). Among the threats to the conservation of this population is cohabitation with livestock, mediated by competition and pathogen transmission (Acevedo et al., 2007; Fonseca et al., 2017; Moço et al., 2014; Walker & Morgan, 2014). The Iberian ibex population at GXTBR lives in sympatry with a large population of extensively managed domestic goats, *Capra hircus* (>16,000 in 2019–INE, 2022), many under organic production systems (Gandra et al., 2016). In these systems, domestic goats are pastured along grazing paths that extend to higher altitudes in spring and summer, eventually overlapping with the Iberian ibex range (Moço et al., 2014). The Iberian ibex population nuclei at GXTBR show different degrees of sympatry with domestic goats, higher in Gerês east and Castro Laboreiro and lower in Serra Amarela and Gerês west nuclei (Moço et al., 2014). Parasites are part of the ecosystem and their impact on host populations might not be readily recognized, but they can affect endangered wildlife health and population dynamics (Walker & Morgan, 2014). This study aimed to assess and compare the excretion of gastrointestinal parasites of Iberian ibex in different degrees of sympatry with domestic goats in GXTBR. The specific aims of this study were to (i) compare the performance of four qualitative diagnostic tests (Mini‐FLOTAC, McMaster, Willis flotation, and natural sedimentation), the first two being also quantitative tests and to (ii) promote the use of these methodologies (molecular host identification coupled with hierarchical models to correct for the imperfect sensibility of the diagnostic tests used in coprological analysis) in other non‐invasive coprological wildlife studies.

## 2 MATERIALS AND METHODS

### 2.1 Sample collection

**FIGURE 1:**
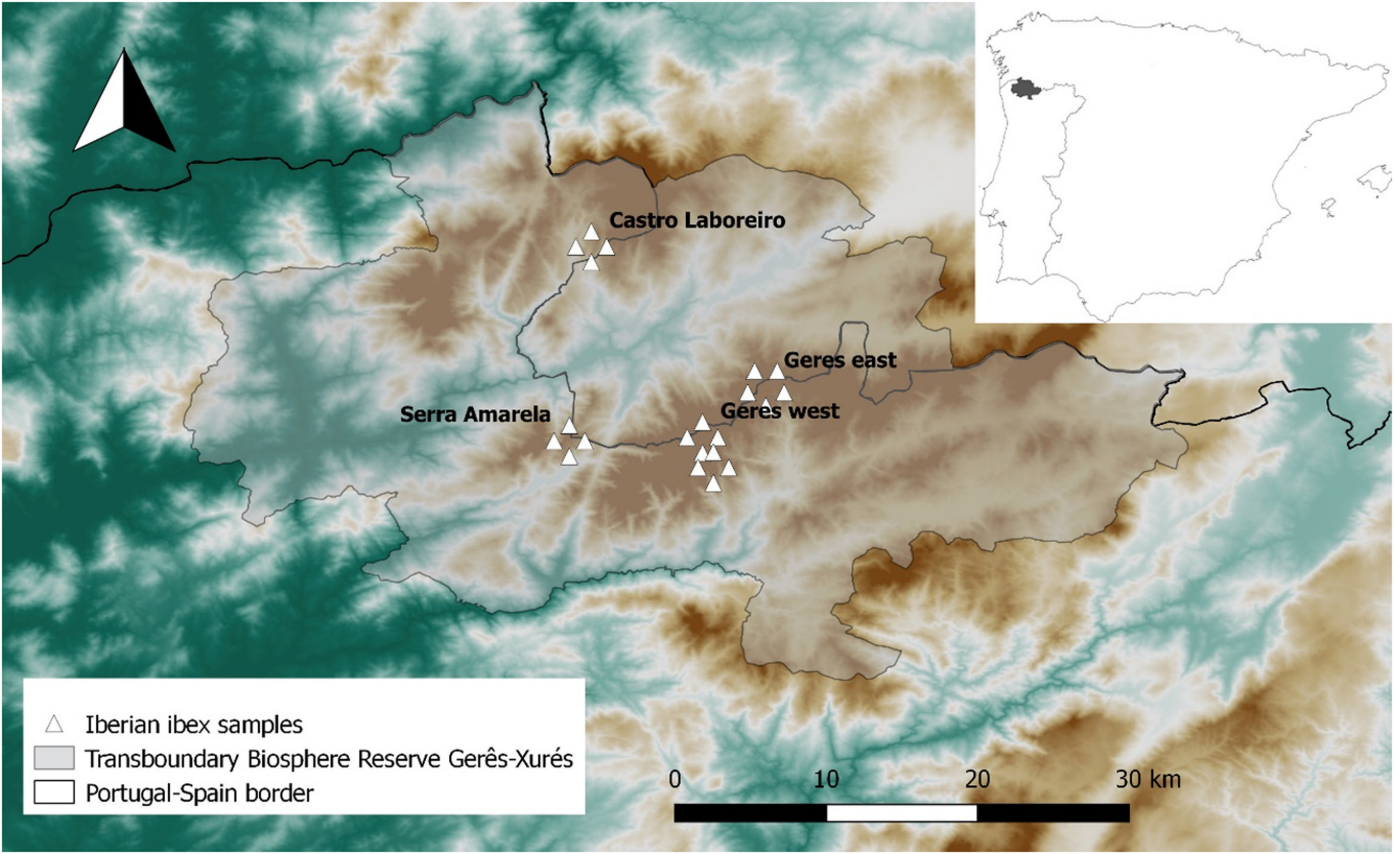
Geographical location of the sample collection sites by population nuclei. Elevation map of the study area with the location of the samples collected by nuclei. Insert: Iberian Peninsula with the Gerês‐Xurés Transboundary Biosphere Reserve highlighted as gray.

Fecal samples were collected in November 2020 along transects on foot targeting the four known population nuclei of Iberian ibex in the GXTBR. The ibex groups (mixed age and sex) were tracked, and 21 pooled samples were collected (Table 1 and Figure 1). Fecal samples were collected whenever Iberian ibex were observed defecating. If such did not occur, the ground where ibex were observed was screened for feces and assessed as reasonably fresh by their texture, moisture, and color. Fresh feces were considered those still wet, shinny, with no cracks on the surface and when pressed between fingers they would not break. Fecal samples of a population nuclei were randomly pooled in 4–8 bags (around 500 g each). The number of individuals and group composition contributing to the pool was variable according to the nucleus: Gerês east 12 individuals (10 females and juveniles and 2 males); Gerês west several groups, around 50–60 individuals total; Serra Amarela a mixed group of 25–30 ibexes; and Castro Laboreiro 2 groups of 15–20 females. Feces were refrigerated (4°C) in zip lock bags to maintain anaerobic conditions for up to 4 weeks until coprological analyses. The collection of samples was performed over 2 weeks and the laboratory analysis was done in chronological order so that the time between collection and analysis was the same for all the samples. Approximately 10 fecal pellets (~10% of the pooled sample) were extracted from each pooled sample 2 to 3 days after collection and stored in 96% ethanol for molecular host species identification.

**TABLE 1:** Number of samples collected and selected for coprological analysis by population nuclei.

Nuclei	Samples collected	Excluded upon molecular analysis		Included in the study
		Failure to extract DNA	Mixed‐species sample	
Gerês west	8	3	0	5
Castro Laboreiro	4	1	0	3
Serra Amarela	4	0	0	4
Gerês east	5	1	1^a^	3
Total	21	5	1	15

*Note*: Molecular analyses of successfully extracted DNA confirmed the field species identification of all the samples included in the study.

^a^ *Capra pyrenaica* – *C. hircus*.

### 2.2 Molecular analysis

All samples underwent molecular analysis to confirm the field species identification and rule out sympatric ruminants (domestic goats, sheep, red, and roe deer). DNA extraction was performed using the EXNA® Tissue DNA KIT (Omega Bio‐tek, Inc., Norcross, GA) and the polymerase chain reaction‐restriction fragment length polymorphism (PCR‐RFLP) for species identification (Fajardo et al., 2009), as previously described (Cardoso et al., 2021). Briefly, cells were obtained from the excrements with a lysis buffer wash (0.1 M Tris–HCl, 0.1 M EDTA, 0.01 M NaCl; 1% N‐lauroyl sarcosine) and DNA was extracted by Omega Bio‐Tek® Protease. PCR settings were: 45 cycles at 95°C for 30 s, 65°C for 40 s, and 72°C for 45 s. Digestions were performed in 10.5 μL containing 3 μL of PCR product, 5 units of MseI endonuclease, and 1 μL CutSmart® Buffer 10× and incubated at 37°C for 3 h. Fragments were visualized with BioRad Universal Hood II using QuantityOne software (BioRad, Hercules, California, USA), after electrophoresis in a 2% TBE agarose gel.

### 2.3 Coprological analysis

Each pooled sample was homogenized, and four diagnostic tests were performed in three replicates. Homogenization was performed by breaking the pellets into powder and mixing. The Mini‐FLOTAC (Cringoli et al., 2017) and modified McMaster (MAFF, 1986) techniques were employed to assess egg/oocyst presence and shedding intensity (eggs and oocysts per gram of feces – EPG and OPG, respectively). The Mini‐FLOTAC technique followed the manufacturer's instructions, mixing 5 g of feces with 45 mL of a saturated sucrose solution (specific gravity 1.2) and using an analytic sensitivity of 5 EPG/OPG (Cringoli et al., 2017). For the modified McMaster, (sensitivity of 50 eggs/g) 2 g of feces and 28 mL of saturated sucrose solution were used (Figueiredo et al., 2020; MAFF, 1986) and both chambers were counted. The same homogenate was then used to perform two other tests, sedimentation, and Willis flotation (Willis, 1921). For that, we performed a combined natural sedimentation/Willis flotation technique for qualitative identification of heavy and light‐weighted eggs, respectively (Ballweber et al., 2014). Eggs and oocysts were assigned to the taxonomic groups' gastrointestinal “Strongylida”, *Moniezia* spp., and eimeriid coccidia using morphological keys (Foreyt, 2002; Thienpont et al., 1979).

### 2.4 Statistical analysis

Hierarchical models separate the detection and state processes. The detection model estimates the detection probability (p) from the replicates of each diagnostic test performed on each sample, this being the sensitivity of the qualitative tests and the probability of detection of one egg or oocyst/gram of feces (quantitative methods). The state models estimate the positivity proportion (site‐occupancy models – ψ) and counts (N‐mixture model—N) corrected for the imperfect detection probability. The true positivity proportion (ψ) was estimated from presence/absence data obtained from the three replicates per sample of each of the four diagnostic tests, using the site‐occupancy model of MacKenzie et al. (2002). Shedding intensity (N) was estimated from egg/oocyst count data (three replicates per sample) obtained from the Mini‐FLOTAC and McMaster techniques using the N‐mixture model of Royle (2004). Statistical analyses were performed in R 3.6.1 using the package ‘unmarked’ (Fiske & Chandler, 2011).

The statistical analyses were performed separately for each of the parasite groups. The explanatory variables were the diagnostic ‘test’ (Mini‐FLOTAC, McMaster, Willis flotation, and natural sedimentation) as observation covariate and ‘nucleus’ (Gerês east, Gerês west, Serra Amarela, and Castro Laboreiro) as site covariates. For each parasite group, the full model was fitted for site‐occupancy and N‐mixture analysis, including all variables in the detection model and the site covariates in the state model using the replicates as response variables. The N‐mixture model was fitted using a negative binomial distribution with *K* = 1500 (Fiske & Chandler, 2011). Models were ranked by their Akaike's Information criterion corrected for small sample size (AICc) (Hurvich & Tsai, 1989) using the package ‘MuMin' (Bartoń, 2019). The most supported model for each parasite group was selected for inference, and models with ΔAIC < 4 are reported in Table 2.

**TABLE 2:** Rank of site‐occupancy and N‐mixture hierarchical models for each parasite group.

Type of model	Parasite group	Model rank	Hierarchical model		*n*Pars	AICc	ΔAICc	Weight
			Detection (*p*)	State (ψ/N)				
Site‐occupancy (~*p* ~ ψ)	Eimeriid coccidia	1	~test	~1	5	184.1	0	0.998
		Full	~test + nuclei	~nuclei	13	264.0	80.0	0
		Null	~1	~1	2	242.4	58.3	0
	Gastro‐intestinal Strongylida	1	~test	~1	5	118.8	0	1
		Full	~test + nuclei	~nuclei	11	208.9	90.1	0
		Null	~1	~1	2	231.3	112.5	0
	*Moniezia* spp.	1	~test	~1	6	83.1	0	0.810
		Null	~1	~1	2	86.2	3.1	0.169
		Full	~test + nuclei	~nuclei	13	168.2	85.1	0
N‐mixture (~*p* ~ N)	Eimeriid coccidia	1	~test + nuclei	~1	7	4339.0	0	1
		Full	~test + nuclei	~nuclei	10	4383.7	44.7	0
		Null	~1	~1	3	4868.6	529.6	0
	Gastro‐intestinal Strongylida	1	~test	~1	4	2833.2	0	0.999
		Full	~test + nuclei	~nuclei	10	2892.6	59.4	0
		Null	~1	~1	3	3398.5	565.4	0
	*Moniezia* spp.	Null	~1	~1	3	284.2	0	0.858
		1	~test	~1	4	288.0	3.82	0.127
		Full	~test + nucleus	~nucleus	10	345.9	61.7	0

Abbreviations: AICc, Akaike's Information Criterion corrected for small sample size; N, Shedding intensity; *n*Pars, number of parameters including intercept; *p*, Detection probability; Weight, model's AICc weight; ΔAICc, difference of AICc with respect to the best model; ψ, Proportion positive.

Site‐occupancy models for the proportion of positivity data. N‐mixture models with negative binomial distribution for shedding intensity data. Models with ΔAICc < 4, full, and null models are shown.

Using the most supported model for each of the parasite groups we then calculated the detection probability of each of the diagnostic tests employed (Table 3). Using the Mini‐Flotac as a reference test we then estimated the proportion of positive and shedding intensity for each parasite group (Table 4).

**TABLE 3:** Estimates of detection probability of each diagnostic test by parasite group.

Type of method	Method	Detection probability (*p*) ± SE		
		Eimeriid coccidia	Gastrointestinal Strongylida	*Moniezia* spp.
Qualitative	Mini‐FLOTAC	0.881 ± 0.050	0.956 ± 0.031	0.382 ± 0.129
	McMaster	0.619 ± 0.075	0.711 ± 0.068	0.064 ± 0.062
	Willis flotation	0.691 ± 0.071	0.978 ± 0.022	0.382 ± 0.129
	Sedimentation	0.071 ± 0.040	0.067 ± 0.037	0 ± 0.001
Quantitative	Mini‐FLOTAC	0.318 ± 0.005	0.028 ± 0.001	0.010 ± 0.002
	McMaster	0.253 ± 0.005	0.060 ± 0.002	0.010 ± 0.002

*Note*: Detection probability ‘*p*’ is the sensitivity of the test (qualitative methods) and the probability of detection of 1 egg or oocyst/g of feces (quantitative methods), with standard errors (SE). Estimates from the most supported models for each parasite group.

**TABLE 4:** Estimates of the proportion positive and shedding intensity for each parasite group in the polled Iberian ibex samples.

Parasite group	Proportion positive (%)		Shedding intensity (OPG/EPG)	
	Hierarchical models	Raw data	Hierarchical models	Raw data
Eimeriid coccidia	0.933 ± 0.064	0.933	703.4 ± 256.8	166.5 ± 126.5
Gastrointestinal Strongylida	1.0 ± 0.001	1.0	1002.1 ± 109.9	31.0 ± 11.6
*Moniezia* spp.	0.349 ± 0.129	0.267	113.7 ± 112.1	1.1 ± 1.7

*Note*: Proportion positive ‘ψ’ and egg/oocyst shedding intensity ‘*N*' (eggs/oocysts per gram) corrected for the imperfect detection probability, with standard error. Estimates from the most supported models for each parasite group using the Mini‐flotac as reference test; raw data refers to the Mini‐flotac test.

## 3 RESULTS

The parasite groups morphologically identified were eimeriid coccidian oocysts, gastrointestinal nematode eggs from the order Strongylida (including *Tricostrongylus* spp. and *Nematodirus* spp.), and *Moniezia* spp. In all the most supported models, except for the shedding intensity of *Moniezia* spp., the detection probability varied with the diagnostic method (Table 2). In the eimeriid coccidia shedding intensity model, detection probability also differed across population nuclei, being higher in Gerês west (0.318 ± 0.005%) than Gerês east (0.164 ± 0.005%), Castro Laboreiro (0.130 ± 0.004%), and Serra Amarela (0.076 ± 0.004%). The detection probability of the qualitative tests (i.e., diagnostic sensitivity) was higher for Mini‐FLOTAC for coccidia, Willis flotation for gastrointestinal Strongylida, and equal for both tests for *Moniezia* spp. (Table 3). The detection probability of the quantitative tests (i.e., probability of detecting 1 egg/oocyst) was higher for Mini‐FLOTAC for coccidia, McMaster for gastrointestinal Strongylida, and equal for *Moniezia* spp. (Table 3).

In all the most supported models, the positivity proportion and shedding intensity estimated using the Mini‐flotac did not differ between population nuclei (Table 4). The estimates of the hierarchical models can be higher than the raw data, particularly when the detection probabilities are low (Table 4).

## 4 DISCUSSION

The combination of molecular host species identification and hierarchical models applied to replicates of diagnostic tests minimized some of the hurdles associated with wildlife non‐invasive parasitological surveys. Assuring that every sample included in the analysis was molecularly assigned the correct host species reduced potential bias in the estimates of prevalence and shedding intensity (Cardoso et al., 2021; Zhang et al., 2011). The morphological characteristic of the samples does not always allow for correct species assessment (Monterroso et al., 2019). In this study, one of the samples field‐assigned to Iberian ibex was also shown to contain fecal DNA of domestic goats, highlighting the strong sympatry of these species in the Gerês east nuclei (Moço et al., 2014). Molecular host species identification thus prevented the inclusion of one mixed‐species sample that could otherwise compromise the results. The main drawback of this approach is that five samples had to be excluded from the analysis for lack of molecular species identification (Table 1). While DNA cannot always be extracted and amplified from fecal samples, success rates >80% are routinely achieved (e.g., Nakamura et al., 2017).

Although the diagnostic tests used in copro‐parasitology are well suited to perform with limited resources, they are also imperfect and hence the estimates they provide can be unreliable. The hierarchical models employed corrected the apparent positivity (proportion positive/total samples) and apparent shedding intensity (OPG and EPG) by the imperfect detection probability of each diagnostic test (MacKenzie et al., 2002; Royle, 2004). The estimates obtained were thus a better approximation of the true positivity proportion and shedding intensity (Lachish et al., 2012; Zanet et al., 2017). This improvement is particularly notorious when the detection probability is low: the estimated positivity proportion of *Moniezia* spp., as well as the estimated shedding intensities of all parasite groups are considerably higher than the raw data. Given the very low detection probabilities estimated by the hierarchical models, the estimated shedding intensities of gastrointestinal Strongylida and *Moniezia* spp are several orders of magnitude higher than the raw data, and the standard errors comparatively lower. Hierarchical models are inherently suited to analyze parasitological wildlife data; nevertheless, they have rarely been used for this purpose (e.g., Zanet et al., 2017).

Pooled samples were used, instead of individual ones, because of the need to perform replicates of the same test in order to fit hierarchical models. Collecting pooled samples guaranteed sufficient fecal material for all the replicates, as individual samples usually do not contain enough fecal material to perform three replicates of three diagnostic tests. Pooled samples also allowed for a population‐level copro‐parasitological assessment that would have otherwise required a much larger number of individual samples. Consequently, the estimates are of the proportion of positivity and not prevalence.

The diagnostic sensitivity of standard copro‐parasitological methods is unknown for many parasite–host systems. In this study, the combination of methodologies estimated the sensitivity of four qualitative coprological tests commonly employed in wildlife parasitological surveys (Abdu et al., 2022). Mini‐FLOTAC showed the highest estimated sensitivity for eimeriid coccidia (0.88), Willis flotation for gastrointestinal Strongylida (0.98), and both tests for *Moniezia* spp. (0.39) (Table 3). Regarding the two quantitative methods employed, Mini‐FLOTAC showed a higher estimated detection probability for eimeriid coccidia (0.32), McMaster for gastrointestinal Strongylida (0.06), and both tests for *Moniezia* spp. (0.01) (Table 3). Broadly similar results were reported in studies comparing Mini‐FLOTAC, McMaster, and other coprological techniques in several animal species (Abdu et al., 2022; Alowanou et al., 2021; Lozano et al., 2021; Rinaldi et al., 2014; Silva et al., 2013). No single copro‐parasitological method performs best for every parasite group. Overall, Mini‐FLOTAC seemed suitable as an all‐purpose copro‐parasitological test and was used here as a reference test to predict the positivity proportion and shedding intensity.

It should be noted that the detection probability estimated refers to the specific protocols used in this study. The McMaster technique employed a lower volume of feces than the Mini‐FLOTAC and increasing it could potentially improve the sensitivity of this method. Due to the logistical constraints of fieldwork in remote areas, samples had to be refrigerated for 4 weeks, potentially decreasing the detection probability (Crawley et al., 2016). One advantage of hierarchical models is that they can correct for this effect of the storage time. In fact, they correct the observed positivity proportion by the lower detection probability, thus yielding estimates potentially closer to the real positivity proportion than if using raw positivity proportion.

Hierarchical models also allowed for assessing the influence of covariates on the detection and state processes. The most‐supported detection models for all host–parasite combinations, except the *Moniezia* spp. shedding intensity, included the effect of the diagnostic test, supporting their different performances (Table 3). Notably, the most supported model for the shedding intensity of eimeriid coccidia also included the effect of the population nuclei, with higher detection probability in the Gerês west nucleus. This observation might be explained by fresher samples collected from this nucleus, which has the highest number of Iberian ibex of all population nuclei, making it easier to find herds and recover freshly voided fecal samples. In our study, feces were collected as fresh as possible upon visual and tactile evaluation; however, rainy weather may alter the perception of feces' morphology and freshness (Agetsuma‐Yanagihara et al., 2017). Future studies employing hierarchical models should assess and grade the samples according to their freshness and include this covariate in the detection models.

No covariates were included in the most‐supported state models, which could show that the proportion of positive and shedding intensity did not differ significantly across population nuclei, as previously reported (Cardoso et al., 2021). We expected to detect an effect on gastrointestinal parasitism of the variable degrees of sympatry with domestic goats between ibex nuclei. However, our study did not achieve enough statistical power (when split between nuclei, the number of pooled samples was between 3 and 5) and therefore no conclusions can be drawn on the differences between nuclei.

The positivity proportion estimates for gastrointestinal Strongylida in Iberian ibex sit at the boundary of the estimable space (1—Table 4). In that situation, the estimated standard errors can be unreliable (Hutchinson et al., 2015). Gastrointestinal Strongylida were detected in 18/19 samples, so the estimated proportion of positive (100%) is realistic. Nevertheless, a larger sample size would be needed to reliably estimate the standard error (Hutchinson et al., 2015).

We report higher *Moniezia* spp. positivity proportion than all the previous studies in Iberian ibex (Cardoso et al., 2021; Luzón et al., 2008; Santiago‐Moreno et al., 2010), although the shedding intensity was similar to that reported in southern Spain (Luzón et al., 2008; Santiago‐Moreno et al., 2010). A similar eimeriid coccidia proportion positive was reported in the GXTBR (Cardoso et al., 2021); however, the shedding intensity reported here is much lower (474 vs. 2441 OPG). This difference might possibly be explained by the seasonality of oocyst shedding in caprines, which peaks after the birth season (Chartier & Paraud, 2012). The modeling approach employed might allow for more precise estimates, and the shedding intensity is higher than reported for Iberian Ibex in southern Spain (Luzón et al., 2008; Santiago‐Moreno et al., 2010). The wet and relatively cold climate of the north‐western Iberian Peninsula (Benayas & Scheiner, 2002) probably explains the observed differences with southern populations of Iberian ibex. The same bioclimatic factors might explain the extremely high positivity proportion of gastrointestinal Strongylida and the shedding intensity similar to (Calero‐Bernal et al., 2020; Cardoso et al., 2021) or higher (Luzón et al., 2008; Santiago‐Moreno et al., 2010) than in other studies.

Our study employed a combination of molecular and statistical methods that improved the estimates of positivity proportion and shedding intensity and allowed us to compare the performance of several diagnostic tests. Molecular confirmation of the host species of non‐invasive samples collected when fecal voiding cannot be observed (e.g., cryptic or rare species or populations) corrects the biases arising from the incorrect specific assignment of samples. Hierarchical models are inherently suited to estimating the sensitivity of the parasitological methods employed and correcting the apparent prevalence and shedding intensity for imperfect sensitivity. Such methodological improvements are critical to enhancing inference in non‐invasive wildlife copro‐parasitological studies.

## AUTHOR CONTRIBUTIONS

**Catarina Fontoura‐Goncalves:** Data curation (equal); formal analysis (equal); investigation (equal); writing – original draft (equal); writing – review and editing (equal). **Érica Portocarrero:** Data curation (equal); formal analysis (equal); investigation (equal); writing – original draft (equal); writing – review and editing (equal). **Ana Oliveira:** Data curation (equal); formal analysis (equal); investigation (equal); writing – original draft (equal); writing – review and editing (equal). **João Lozano:** Formal analysis (equal); methodology (equal); validation (equal); writing – review and editing (equal). **Laura Rinaldi:** Funding acquisition (equal); resources (equal); writing – review and editing (equal). **Giuseppe Cringoli:** Funding acquisition (equal); resources (equal); writing – review and editing (equal). **Luís Madeira de Carvalho:** Conceptualization (equal); data curation (equal); funding acquisition (equal); methodology (equal); project administration (equal); resources (equal); supervision (equal); validation (equal); writing – review and editing (equal). **Nuno Santos:** Conceptualization (equal); data curation (equal); formal analysis (equal); funding acquisition (equal); investigation (equal); methodology (equal); project administration (equal); resources (equal); supervision (equal); validation (equal); visualization (equal); writing – original draft (equal); writing – review and editing (equal).

## CONFLICT OF INTEREST STATEMENT

On behalf of all authors, the corresponding author states that there is no conflict of interest.

## Data Availability

Data are available at Dryad DOI: https://doi.org/10.5061/dryad.2547d7wwp.
